# Renoprotection Provided by Additional Diuretic Treatment in Partially Nephrectomized Ren-2 Transgenic Rats Subjected to the Combined RAS and ET_A_ Blockade

**DOI:** 10.3389/fphys.2019.01145

**Published:** 2019-09-18

**Authors:** Ivana Vaněčková, Silvie Hojná, Zdenka Vernerová, Michaela Kadlecová, Hana Rauchová, Elzbieta Kompanowska-Jezierska, Zdeňka Vaňourková, Luděk Červenka, Josef Zicha

**Affiliations:** ^1^Department of Experimental Hypertension, Institute of Physiology, Czech Academy of Sciences, Prague, Czechia; ^2^Department of Pathology, Third Faculty of Medicine, Charles University, Prague, Czechia; ^3^Department of Renal and Body Fluid Physiology, Mossakowski Medical Research Centre, Polish Academy of Sciences, Warsaw, Poland; ^4^Institute for Clinical and Experimental Medicine, Prague, Czechia

**Keywords:** hydrochlorothiazide, renoprotection, nephrectomy, atrasentan, chronic kidney disease, losartan, trandolapril

## Abstract

**Objective:**

Our previous study in heterozygous Ren-2 transgenic rats (TGR) demonstrated that long-term treatment with endothelin receptor A (ET_A_) blocker atrasentan added to the renin-angiotensin system (RAS) blockade had renoprotective effects in a model of chronic kidney disease (CKD) induced by partial nephrectomy. Since ET_A_ blockade is known to cause edema, we were interested whether diuretic treatment added to this therapy would be beneficial.

**Design and Methods:**

Partial nephrectomy (NX) was performed at the age of 3 months in TGR rats which were subjected to: (i) RAS blockade alone (angiotensin receptor blocker losartan and angiotensin converting enzyme inhibitor trandolapril), (ii) combined RAS (losartan and trandolapril) and ET_A_ receptor blockade (atrasentan), or (iii) diuretic (hydrochlorothiazide) added to the combined RAS + ET_A_ blockade for 50 weeks following NX.

**Results:**

At the end of the study systolic blood pressure and cardiac hypertrophy were similarly decreased in all treated groups. Survival was significantly improved by ET_A_ receptor blockade added to RAS blockade with no further effects of diuretic treatment. However, additional diuretic treatment combined with RAS + ET_A_ blockade decreased body weight and had beneficial renoprotective effects – reductions of both kidney weight and kidney damage markers. Proteinuria gradually increased in rats treated with RAS blockade alone, while it was substantially lowered by additional ET_A_ blockade. In rats treated with additional diuretic, proteinuria was progressively reduced throughout the experiment.

**Conclusion:**

A diuretic added to the combined RAS and ET_A_ blockade has late renoprotective effects in CKD induced by partial nephrectomy in Ren-2 transgenic rats. The diuretic improved: renal function (evaluated as proteinuria and creatinine clearance), renal morphology (kidney mass, glomerular volume), and histological markers of kidney damage (glomerulosclerosis index, tubulointerstitial injury).

## Introduction

Chronic kidney disease (CKD) is often associated with hypertension, diabetes and obesity. Due to its increasing occurrence, it represents an important burden for a healthcare system. Since the symptoms are often unrecognized, it is usually found later in life as it progresses to end-stage renal disease (Zhong et al., 2017). Moreover, as the world population is living longer, the prevalence of CKD increases. The most widely used therapy of CKD, the “gold standard,” is provided by the inhibition of the renin-angiotensin system (RAS) exerting both antihypertensive and nephroprotective effects (Raij, 2005). However, this therapy is not able to halt the progression of CKD to end-stage renal disease (ESRD) and, therefore, new additive/combination therapies are strongly needed (Breyer and Susztak, 2016; Komers and Plotkin, 2016).

One of the treatment possibilities is the blockade of endothelin receptor A (ET_A_) since the activation of these receptors with endothelin-1 (Yanagisawa et al., 1988; Davenport et al., 2016, 2018) is associated with inflammation, proliferation, fibrosis and renal cell injury (including podocyte injury) which ultimately leads to proteinuria. Many experimental as well as clinical studies indicated favorable potential of endothelin receptor antagonists (ERAs) – “sentans” – in the treatment of CKD especially due to their renoprotective capacities (for review see Kohan and Pollock, 2013; Kohan and Barton, 2014; Culshaw et al., 2015; Czopek et al., 2016; Vaněčková et al., 2018; Dhaun and Webb, 2019).

Ren-2 transgenic rats are a model of angiotensin II-dependent hypertension associated with an inappropriately activated RAS due to the insertion of mouse renin-2 gene into the Hannover Sprague-Dawley rats (Mullins et al., 1990). In combination with subtotal nephrectomy (Morrison, 1962) severe renal impairment is induced not only due to a markedly activated RAS but also due to highly elevated endothelin levels (Vaněčková et al., 2012; Čertíková Chábová et al., 2014; Sedláková et al., 2017). Thus, this model is of critical importance since it combines high blood pressure and activated renin-angiotensin and endothelin systems, which contribute to the progression of CKD to ESRD.

Our studies in this particular model of CKD (5/6 NX in Ren-2 transgenic rats) revealed that the chronic sole endothelin receptor A blockade with atrasentan improved survival rate, prevented the development of enhanced hypertension following subtotal nephrectomy, and attenuated end-organ damage (Vaněčková et al., 2012). Moreover, if chronic atrasentan treatment was combined with RAS blockade (maximal doses of angiotensin receptor blocker losartan and angiotensin converting enzyme inhibitor trandolapril), it exerted beneficial antiproteinuric effects. However, these effects were seen only after a very long treatment (44 weeks) (Čertíková Chábová et al., 2014), while they were not demonstrated after a shorter period (20 weeks) (Vaněčková et al., 2012). The beneficial effects of additional ET_A_ blockade were also lost when the treatment was postponed to the phase of established CKD, i.e., when the therapy was started 6 weeks after the induction of CKD by the subtotal nephrectomy (Sedláková et al., 2017).

The benefits of the therapy with endothelin receptor A antagonists are limited not only by their tolerability profile, but also by fluid retention which led to the termination of several big studies, including ASCEND (Mann et al., 2010) and RADAR (de Zeeuw et al., 2014). The final results of the SONAR clinical trial (Heerspink et al., 2019) demonstrated positive effect of atrasentan in the reduction of renal events in patients with type 2 diabetes and CKD although fluid retention in a number of patients still occurred. Moreover, the DUET study (Komers et al., 2017) in patients with focal segmental glomerulosclerosis (FSGS) (Trachtman et al., 2018) demonstrated better renoprotection by sparsentan (dual RAS and ET_A_ blocker) compared to irbesartan following 8 weeks of treatment: sparsentan being well tolerated and safe.

Apart from the liver toxicity and anemia the most significant side effect associated with endothelin receptor antagonists is fluid retention. A possible mechanism is the redistribution of body fluids within the body due to an increase in vascular permeability following the activation of ET_B_ receptors during ET_A_ receptor blockade (Vercauteren et al., 2017). Since the body weight (BW) gain and peripheral edema have been encountered to a certain degree with all endothelin receptor A antagonists, the use of diuretics to circumvent this problem was considered to be reasonable. Additionally, diuretics are inexpensive and efficient antihypertensives which can be used even in the advanced stages of CKD. Specifically, the concomitant use of thiazides (in combination with the inhibitors of the RAS) is now being revised in the treatment of this disease (Sinha and Agarwal, 2015) based on the clinical studies showing their positive effects on BP and proteinuria (Cirillo et al., 2014; Morales et al., 2015; Fuwa et al., 2016). However, there are almost no experimental data showing the positive effects of ET_A_ receptor antagonists in combination with diuretics. The only information available is that chlorthalidone combined with atrasentan exerted an antiproteinuric effect in Dahl rats fed a high fat/high salt diet which is a model of a metabolic syndrome (Jin et al., 2014).

Therefore, in the present study, we hypothesized whether the use of a diuretic alleviates the negative side effects of ET_A_ receptor blockade leading to fluid retention and whether the use of hydrochlorothiazide is beneficial in a model of CKD induced by partial nephrectomy in hypertensive Ren-2 transgenic rats.

## Materials and Methods

### Animals

Adult male heterozygous (mRen-2)27 transgenic (TGR) rats were housed at 23°C under a 12 h light/dark cycle, fed an Altromin diet (0.45% NaCl) and given tap water *ad libitum*. Partial nephrectomy (removal of the right kidney and both poles of the contralateral kidney) was performed in rats under anesthesia (tiletamine + zolazepam, Virbac SA, Carros Cedex, France, 8 mg/kg; and xylazine, Spofa, Czechia, 4 mg/kg intramuscularly), as described previously (Vaněčková et al., 2012; Čertíková Chábová et al., 2014) at the age of 3 months.

All the procedures and experimental protocols were approved by the Ethical Committee of the Institute of Physiology, Czech Academy of Sciences, and conformed to the European Convention on Animal Protection and Guidelines on Research Animal Use.

### Chronic Treatment in Subtotally Nephrectomized TGR

We used three different therapies: (i) RAS blockade alone – angiotensin receptor blocker losartan (10 mg/kg/day; Lozap, Zentiva, Czechia) and angiotensin converting enzyme inhibitor trandolapril (0.6 mg/kg/day; Gopten, Abbott, Germany), (ii) combined RAS (losartan and trandolapril) and ET_A_ receptor blockade (atrasentan – 5 mg/kg/day; ABT-627, a generous gift from AbbVie, IL, United States), and (iii) diuretic hydrochlorothiazide (6 mg/kg/day; Léčiva, Czechia) given with the combined RAS + ET_A_ blockade. All these treatments started immediately after nephrectomy and lasted 50 weeks. The drug dosages were selected on the basis of previous studies (Vaněčková et al., 2012; Arias et al., 2013; Čertíková Chábová et al., 2014). The concentration of atrasentan was adjusted weekly according to the actual water intake to achieve a constant dose of atrasentan (5 mg/kg/day); this dosage was previously found to effectively block ET_A_ receptors (Vaněčková et al., 2012, 2018).

The following experimental groups were investigated:

1. Sham-operated TGR + water (*n* = 12).
2. 5/6 NX TGR + water (*n* = 18).
3. 5/6 NX TGR + RAS blockade (*n* = 10).
4. 5/6 NX TGR + RAS blockade + ET_A_ blockade (*n* = 10).
5. 5/6 NX TGR + RAS blockade + ET_A_ blockade + diuretic (*n* = 10).

Systolic BP, diastolic BP, and heart rate were measured by a direct cannulation of the carotid artery under isoflurane anesthesia (1.5% isoflurane) using a pressure transducer and a multichannel recorder (ADInstruments, Bella Vista, Australia) at the end of the experiment. Body weight and survival were monitored throughout the experiment. At weeks 1, 5, 9, 13, 17, 20, 30, 40 and 50, the animals were placed in individual metabolic cages for 24-h urine collection and proteinuria and creatinine excretion were determined. At these same measurement points, blood samples were withdrawn for the determination of creatinine concentration in plasma. This approach is regularly used and validated in our laboratory (Vaněčková et al., 2012; Čertíková Chábová et al., 2014). Urinary protein was measured using the Folin method with bovine serum albumin as a standard (Lowry et al., 1951). Plasma creatinine was measured by a FUJI DRI-CHEM analyzer using appropriate slides for creatinine CRE-P III (FUJIFILM Corp., Tokyo, Japan). Urine creatinine was determined using a Liquick Cor-CREATININE kit that is based on the modified Jaffe’s method, without deprotenization (PZ CORMAY S.A., Poland). In an alkaline solution picrate reacts with creatinine to form a yellow-red 2,4,6-trinitrocyclohexadienate. The color intensity, measured by a photometer at 500 nm, is proportional to the creatinine concentration. Clearance of creatinine was calculated using a standard formula and was normalized per body weight. At the end of the study, the kidney and heart were weighed. The central part of the left kidney was always used to assess renal glomerular damage.

### Histological Examination

The kidneys for histological analysis were fixed in 4% formaldehyde, dehydrated and embedded in paraffin. The sections stained with hematoxylin-eosin and PAS (periodic acid, for Schiff reaction) were examined and evaluated in a blind-test fashion. Fifty glomeruli in each kidney were examined on a semi-quantitative scale as described previously (Saito et al., 1987): grade 0, all glomeruli normal; grade 1, sclerotic area up to 25% (minimal sclerosis); grade 2, sclerotic area 25 to 50% (moderate sclerosis); grade 3, sclerotic area 50 to 75% (moderate-to-severe sclerosis); and grade 4, sclerotic area 75 to 100% (severe sclerosis). The glomerulosclerosis index (GSI) was calculated using the following formula: GSI = [(1 × *n*1) + (2 × *n*2) + (3 × *n*3) + (4 × *n*4)]/(*n*0 + *n*1 + *n*2 + *n*3 + *n*4), where *nx* is the number of glomeruli in each grade of glomerulosclerosis. Renal cortical tubulointerstitial injury was evaluated on the basis of inflammatory cell infiltration, tubular atrophy, and interstitial fibrosis, using the semi-quantitative scoring method: for tubular atrophy: grade 0, no atrophy; grade 1, mild (<25% of the tubuli atrophic); grade 2, moderate (25–50% of the tubuli atrophic); and grade 3, severe (>50% of the tubuli atrophic) (Nakano et al., 2008). Inflammatory infiltrate and interstitial fibrosis were graded as mild (grade 1), moderate (grade 2) and severe (grade 3). The lesions were assessed in at least 30 random and non-overlapping fields in the renal cortex. Morphometric evaluation of the cross-sectional area of glomeruli (glomerular area) was performed on the same kidney sections that were examined for morphological changes with the method employed in our previous study (Rakusan et al., 2010), using the Nikon NIS-Elements AR 3.1 morphometric program (Nikon, Tokyo, Japan).

### Statistics

The data are presented as the means ± SEM. Statistical analysis was done by Student’s *t*-test, Wilcoxon’s signed-rank test for unpaired data, or one-way analysis of variance (ANOVA) when appropriate, using the Graph-Pad Prism software (Graph Pad Software, San Diego, CA, United States), and ANOVA for repeated measurements, followed by Student–Newman–Keuls test performed for the analysis within groups, when appropriate. Comparison of the survival curves was performed using a log-rank (Mantel–Cox) test followed by a Gehan–Breslow–Wilcoxon test. In order to obtain reliable data regarding the effects of treatment regimens on the survival rate, high initial *n* values were used to enable valid comparison of the long-term survival rate. These initial *n* values were estimated by statistical power analyses method developed by Cohen (2013).

## Results

**FIGURE 1:**
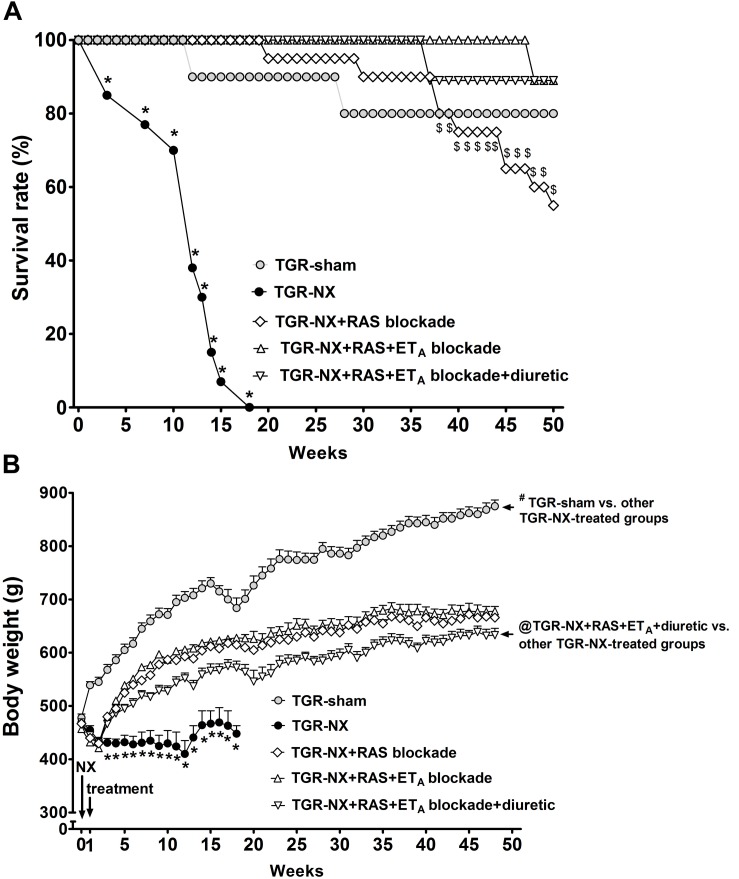
The effect of diuretic treatment added to combined RAS and ET_A_ blockade on survival and body weight. Survival rate **(A)** and body weight **(B)** in sham-operated TGR (TGR-sham), nephrectomized TGR (TGR-NX) untreated receiving water or treated with the renin-angiotensin system (RAS) blockade alone (TGR-NX + RAS blockade), combined RAS + ET_A_ blockade (TGR-NX + RAS + ET_A_ blockade) or diuretic given with the combined RAS + ETA blockade (TGR-NX + RAS + ET_A_ blockade + diuretic). # *P* < 0.05 TGR-sham vs. other TGR-NX-treated groups, $ *P* < 0.05 TGR-NX + RAS treated vs. other TGR-NX-treated groups, @ *P* < 0.05 TGR-NX + RAS + ETA + diuretic vs. other TGR-NX-treated groups, ^∗^*P* < 0.05 untreated TGR-NX group compared with all other treated TGR-NX groups at the same time point.

While 80% of the sham-operated TGR survived until the end of the experiment, untreated TGR-NX rats began to die at week 4 and no TGR-NX rat survived 18 weeks following nephrectomy (Figure 1A). In contrast, RAS blockade alone substantially improved survival to the point that 55% of the animals were alive at the end of the study (52 weeks after surgery). Moreover, combined RAS + ET_A_ blockade without or with additional diuretic further increased the survival of these animals, reaching almost 90% at the end of the study.

Partial nephrectomy resulted in a substantial reduction of body weight gain in all groups but the treatments used in our experiments enabled a significant, subsequent increase in body weight, although the body weight in all treated groups of TGR-NX animals was still lower compared to the sham-operated TGR rats (Figure 1B). The body weight of partially nephrectomized rats, which were treated with a diuretic given with the combined RAS + ET_A_ blockade, was significantly lower than in rats treated with RAS blockade alone or with combined RAS + ET_A_ blockade during the study, suggesting reduced fluid retention.

**FIGURE 2:**
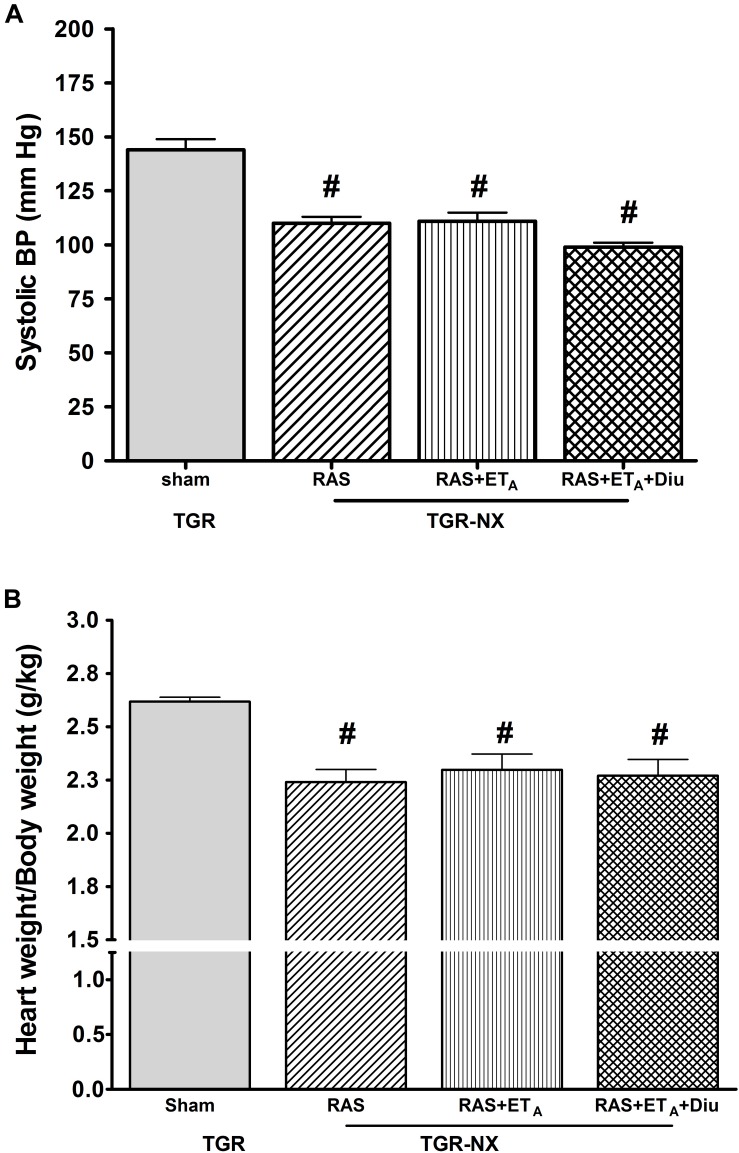
The effect of diuretic treatment added to combined RAS and ET_A_ blockade on SBP and cardiac hypertrophy. Systolic blood pressure **(A)** and cardiac hypertrophy **(B)** in sham-operated heterozygous Ren-2 transgenic rats (TGR-sham), nephrectomized TGR (TGR-NX) treated with the renin-angiotensin system blockade alone (RAS), combined RAS + ET_A_ blockade (RAS + ET_A_); or diuretic given with the combined RAS + ET_A_ blockade (RAS + ET_A_ + Diu). # *P* < 0.05 TGR-sham vs. other TGR-NX-treated groups, Diu – diuretic.

Although systolic BP was similarly reduced by all three therapies showing the prevailing BP effect of RAS blockade, there was a mild tendency for a further drop of BP in diuretic-treated rats (110 ± 3 mm Hg in RAS blockade alone; 111 ± 4 mm Hg in combined RAS + ET_A_ blockade; 99 ± 2 mm Hg in combined RAS + ET_A_ blockade + diuretic) (Figure 2A). The sham-operated TGR exhibited distinct cardiac hypertrophy evaluated as heart weight/body weight ratio (2.62 ± 0.02 g/kg BW) (Figure 2B). Consistent with the normalization of BP following the treatments, relative cardiac weight was similarly decreased in all treated groups (2.24 ± 0.06 g/kg BW in RAS blockade alone; 2.29 ± 0.08 g/kg BW in combined RAS + ET_A_ blockade; 2.27 ± 0.08 g/kg BW in combined RAS + ET_A_ blockade + diuretic). Similarly, there were no differences in cardiac or left ventricular weights related to tibial length among TGR-NX animals subjected to the above treatments (data not shown).

**FIGURE 3:**
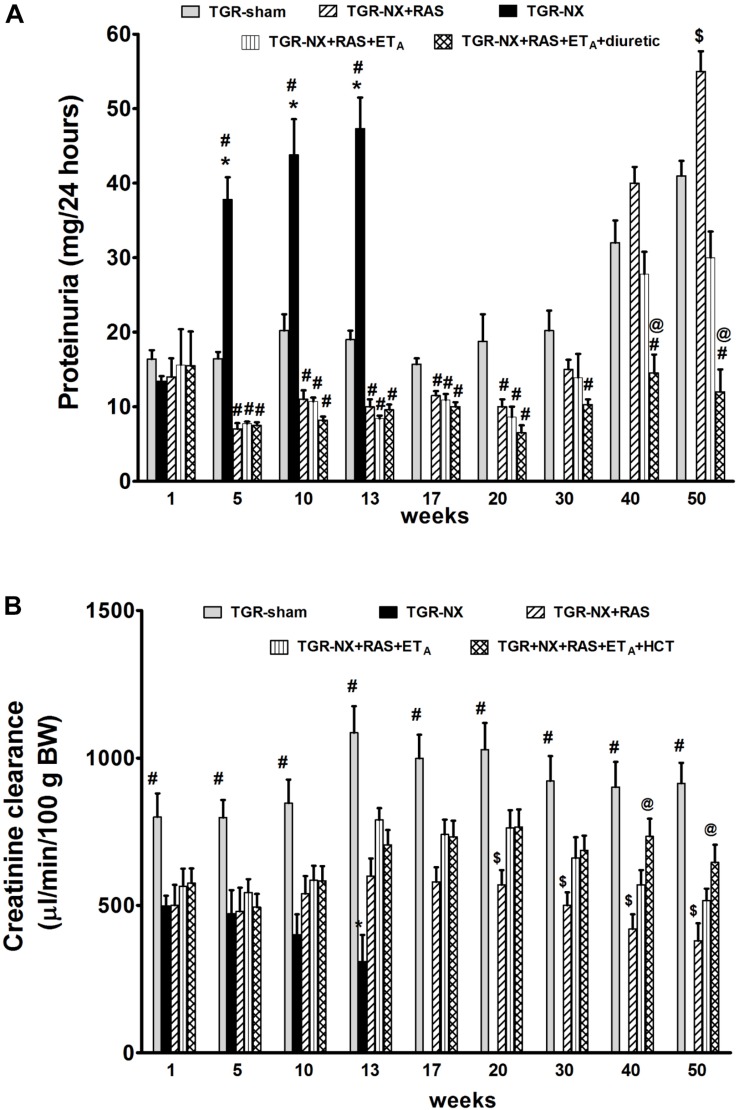
The effect of diuretic treatment added to combined RAS and ETA blockade on proteinuria and creatinine clearance. Proteinuria **(A)** and creatinine clearance **(B)** in sham-operated TGR (TGR-sham), nephrectomized TGR (TGR-NX) untreated receiving water or treated with the renin-angiotensin system (RAS) blockade alone (TGR-NX + RAS), combined RAS + ET_A_ blockade (TGR-NX + RAS + ET_A_), or diuretic given with the combined RAS + ET_A_ blockade (TGR-NX + RAS + ET_A_ blockade + diuretic). # *P* < 0.05 TGR-sham vs. other TGR-NX-treated groups, $ *P* < 0.05 TGR-NX + RAS treated vs. other TGR-NX-treated groups, @ *P* < 0.05 TGR-NX + RAS + ET_A_ + diuretic vs. other TGR-NX-treated groups, ^∗^*P* < 0.05 untreated TGR-NX group compared with all other treated TGR-NX groups at the same time point.

There was only a gradual increase of proteinuria in sham-operated TGR during the study (16.4 ± 1.2 mg/24 h at week 1 vs. 41 ± 2 mg/24 h at week 50) (Figure 3A). In contrast, proteinuria in untreated TGR-NX rats rose steeply between weeks 1 and 5 after nephrectomy (13.4 ± 0.7 mg/24 h vs. 37.8 ± 3.0) and remained high until the death of the animals (47.3 ± 4.2 mg/24 h). On the other hand, all three treatments slightly attenuated proteinuria during the first month of therapy (week 1 vs. week 5: 14 ± 2.5 mg/24 h vs. 7 ± 0.8 mg/24 h in RAS blockade alone; 15.6 ± 4.8 mg/24 h vs. 7.7 ± 0.3 mg/24 h in the combined RAS + ET_A_ blockade; and 15.5 ± 4.6 mg/24 h vs. 7.5 ± 0.4 mg/24 h in the combined RAS + ET_A_ blockade + diuretic) and proteinuria was significantly lowered when compared with sham-operated TGR (16.5 ± 0.9 mg/24 h). The treatment with RAS blockade alone was able to prevent the dramatic increase in proteinuria observed in untreated TGR-NX rats until week 20 after nephrectomy (10.2 ± 1.1 mg/24 h in TGR-NX + RAS at week 20 vs. 47.3 ± 4.2 mg/24 h in TGR-NX at week 13). However, after 30 weeks following nephrectomy a late, gradual increase in proteinuria occurred in the TGR-NX + RAS group, reaching 55.3 ± 2.7 mg/24 h at week 50. In contrast, additional ET_A_ blockade partially reversed this trend and diuretic treatment given with the combined RAS + ET_A_ blockade maintained proteinuria at low levels throughout the entire experiment (30 ± 2.5 mg/24 h in RAS + ET_A_ blockade and 12.4 ± 3 mg/24 h in RAS + ET_A_ blockade + diuretic at week 50) (Figure 3A).

Creatinine clearance was stable in the sham-operated TGR rats during the entire experiment (Figure 3B). By contrast, creatinine clearance dramatically decreased after partial nephrectomy in all groups. In untreated animals, it rapidly declined with time, while all three treatments were able to partially reverse this drop of creatinine clearance. Moreover, creatinine clearance gradually decreased in the TGR-NX rats with RAS blockade alone, while additional ET_A_ blockade partly prevented this decrease. It is important to emphasize that the beneficial effects of diuretic added to RAS + ET_A_ blockade on glomerular filtration were observed especially in the late phase of the study (weeks 40 to 50) (Figure 3B).

**FIGURE 4:**
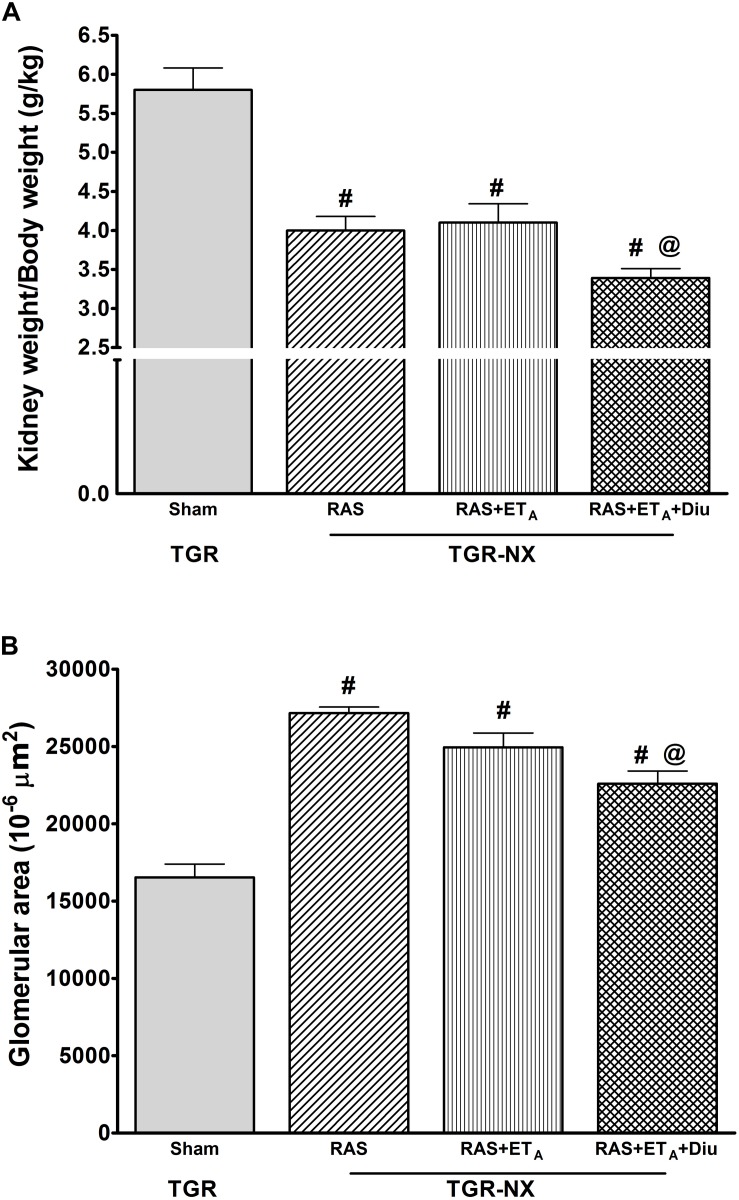
The effect of diuretic treatment added to combined RAS and ET_A_ blockade on relative kidney weight and glomerular area. Relative kidney weight **(A)** and glomerular area **(B)** in sham-operated heterozygous Ren-2 transgenic rats (TGR-sham), nephrectomized TGR (TGR-NX) treated with the renin-angiotensin system blockade alone (RAS), combined RAS + ET_A_ blockade (RAS + ET_A_); or diuretic given with the combined RAS + ET_A_ blockade (RAS + ET_A_ + Diu). # *P* < 0.05 TGR-sham vs. other TGR-NX-treated groups, Diu – diuretic. # *P* < 0.05 TGR-sham vs. other TGR-NX-treated groups, @ *P* < 0.05 TGR-NX + RAS + ET_A_ + diuretic vs. other TGR-NX-treated groups.

**FIGURE 5:**
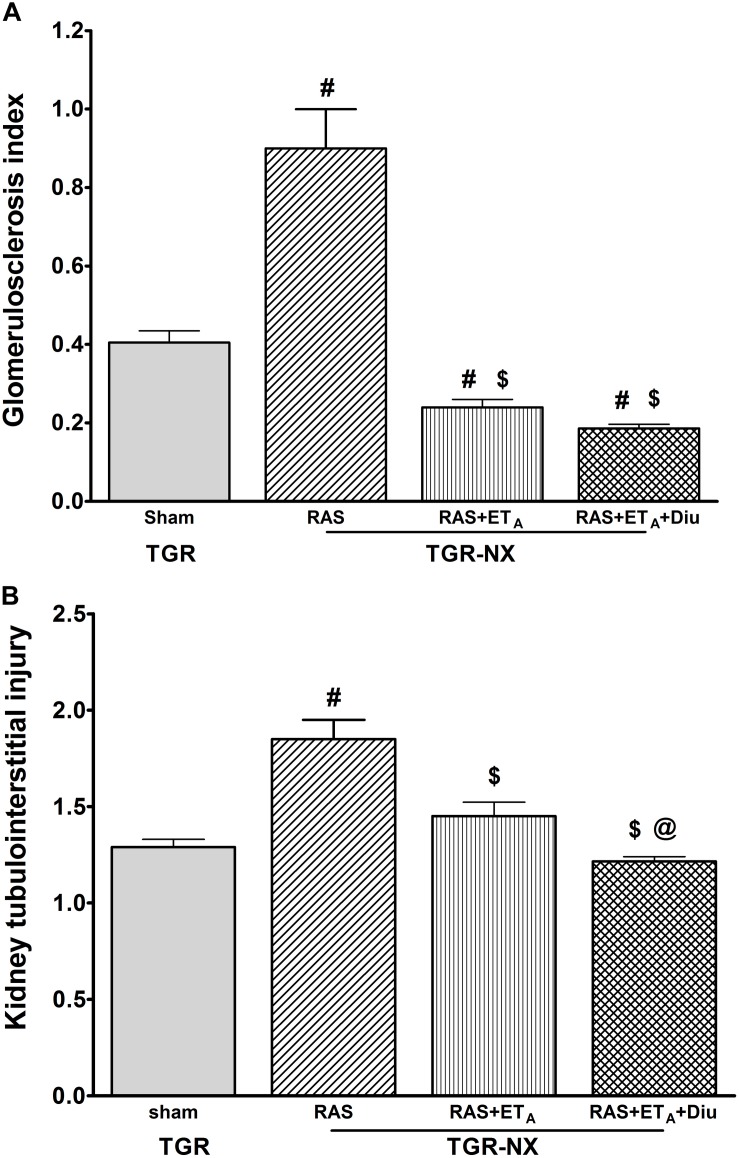
The effect of diuretic treatment added to combined RAS and ET_A_ blockade on histological markers of renal damage. Glomerulosclerosis index **(A)** and kidney tubulointerstitial injury **(B)** in sham-operated heterozygous Ren-2 transgenic rats (TGR-sham), nephrectomized TGR (TGR-NX) treated with the renin-angiotensin system blockade alone (RAS), combined RAS + ET_A_ blockade (RAS + ET_A_); or diuretic given with the combined RAS + ET_A_ blockade (RAS + ET_A_ + Diu). Diu – diuretic # *P* < 0.05 TGR-sham vs. other TGR-NX-treated groups, $ *P* < 0.05 TGR-NX + RAS treated vs. other TGR-NX-treated groups, @ *P* < 0.05 TGR-NX + RAS + ET_A_ + diuretic vs. other TGR-NX-treated groups.

**FIGURE 6:**
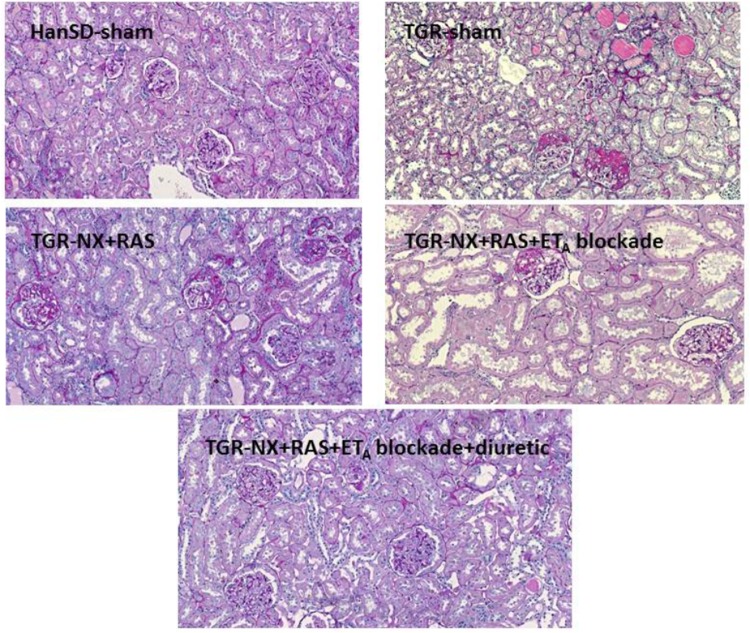
Representative images of renal histology. Renal parenchyma without morphological alterations in control sham-operated HanSD rats (HanSD-sham). Extensive secondary focal segmental glomerulosclerosis and small zone of atrophic tubules (the upper right part) in sham-operated heterozygous Ren 2 transgenic rats (TGR-sham). RAS blockade only partially improved morphology of renal parenchyma; focal segmental glomerulosclerosis persists as well as glomerular enlargement in nephrectomized TGR (TGR-NX + RAS). Combined RAS and ETA blockade substantially atenuated renal injury in nephrectomized TGR; glomeruli contain segmental tuft sclerosis only sporadically (TGR-NX + RAS + ET_A_ blockade). Treatment with diuretic added to the combined RAS+ETA blockade most significantly protected renal parenchyma from injury in nephrectomized TGR (TGR-NX + RAS + ET_A_ blockade + diuretic). Periodic acid-Schiff staining. Magnification 200×.

The relative kidney weights (evaluated as kidney weight/body weight) (Figure 4A) in all treated nephrectomized TGR groups were significantly lower than those in sham-operated TGR (4.0 ± 0.18 g/kg BW in RAS blockade alone; 4.1 ± 0.24 mg/kg BW in the combined RAS + ET_A_ blockade and 3.39 ± 0.12 g/kg BW in RAS + ET_A_ blockade + diuretic vs. 5.8 ± 0.28 g/kg BW in TGR-sham). It is important to note that the addition of a diuretic strongly reduced kidney enlargement which is a crucial process underlying CKD progression. A similar effect was also observed using histological examination – the glomerular area (Figure 4B) of rats treated with additional hydrochlorothiazide was substantially smaller in comparison with animals treated with RAS blockade alone or with the combined RAS + ET_A_ blockade. The RAS blockade alone was not able to diminish the renal alterations caused by partial nephrectomy (glomerulosclerosis index and tubulointerstitial injury) (Figure 5) to a substantial degree but the addition of ET_A_ receptor blocker atrasentan to RAS therapy significantly attenuated both parameters. Finally, diuretic treatment caused a further drop in the glomerulosclerosis index and kidney tubulointerstitial injury. Representative kidney slices stained with periodic acid-Schiff (PAS) are depicted in Figure 6.

## Discussion

This is the first study showing the beneficial effects of a diuretic added to a combination of the RAS inhibitors and endothelin receptor A antagonist atrasentan in a model of CKD which was induced by subtotal nephrectomy in hypertensive heterozygous Ren-2 transgenic rats. While the ET_A_ receptor blockade added to the RAS blockade improved survival, decreased proteinuria and reduced indices of renal injury (glomerulosclerosis index and tubulointerstitial injury), the addition of hydrochlorothiazide had further profound effects on the proteinuria and creatinine clearance, and substantially reduced the kidney weight and the glomerular area. However, the beneficial effects of diuretic therapy on proteinuria and creatinine clearance were evident only after a very long treatment (40–50 weeks). This is consistent with our previous study in which we demonstrated no additional renoprotective effects of ET_A_ receptor blockade combined with RAS blockade if the treatment lasted only 20 weeks (Vaněčková et al., 2012). In contrast, the positive effects, observed both in the earlier study (Čertíková Chábová et al., 2014) and in this one, were seen after the same treatment which lasted 40–50 weeks. Considering the importance for clinical practice, one should bear in mind that such a lifelong treatment resembles the antihypertensive treatment which is provided to patients for many decades. This should be considered in the design of clinical studies to ensure that they are of sufficient duration (i.e., adequately prolonged) in order to observe the benefits of such therapies in patients. It is evident that the translation of experimental results into clinical practice is difficult and sometimes not successful as has been described with the dual RAS blockade in CKD (Čertíková Chábová and Červenka, 2017).

It seems that there is a critical moment, i.e., at 20–30 weeks of treatment, when the sole RAS inhibition fails to be a sufficient therapy of CKD and additional therapeutic interventions are necessary. Such decreased efficiency of the treatment with RAS inhibitors and therefore the necessity of additional therapy were observed not only in the present study but also in the previous one (Čertíková Chábová et al., 2014). Moreover, similar findings of a substantial increase in proteinuria associated with an increased mortality after 30 weeks of treatment were also observed in TGR rats in which the treatment was postponed to the established phase of CKD (Sedláková et al., 2017). The results of our studies suggest some structural or functional changes in the kidney that are sensitive to additional treatments. Unfortunately, there are no other experimental studies with such long treatments that could enable us to discuss this idea. Therefore, further studies are needed to elucidate this issue.

As has been already discussed, the animals treated with RAS blockade alone started to die dramatically after 30 weeks of therapy. Although we did not determine the cause of death in rats subjected to partial nephrectomy in this particular study, the comparison of survival curve with the development of proteinuria or creatinine clearance indicates that the early death of untreated TGR-NX rats at 10–15 weeks after surgery as well as the late death of TGR-NX rats subjected to RAS blockade alone at 35–50 weeks after surgery was associated with a major reduction of GFR and a substantial increase of albuminuria. Thus, renal failure rather than heart failure might be the cause of their deaths. Nevertheless, a previous study of our colleagues, Kujal and Vernerová (unpublished results), who analyzed the cause of deaths following 5/6 nephrectomy demonstrated that about 50% of animals subjected to 5/6 nepherectomy died from heart failure during the 16 weeks of their study. The additional ET_A_ receptor therapy (with or without a diuretic) substantially increased survival, suggesting the importance of ET_A_ therapy. However, since we did not include the groups with diuretic added to RAS blockade (without ET_A_ receptor blockade) and diuretic added to ET_A_ receptor blockade (without RAS blockade), we are not able to determine which particular blockade would be more efficient. The reason for why we did not include these groups was the fact that the blockade of the RAS is the standard therapy of CKD; so that all therapies used in our study were based on RAS blockade. Nevertheless, on the basis of the better nephroprotective effects of therapy with a diuretic added to the combined RAS + ET_A_ blockade especially at the final stages of our study, one would anticipate that the survival in this particular group would improve significantly if the study were to be further prolonged.

Creatinine clearance in sham-operated TGR was 800–1000 μl/min/100 g BW, which was reduced to 500 after NX with a further drop to 300 at week 13. Similarly low value (380 μl/min/100 g BW) was found in TGR-NX treated with RAS blockade at week 50 after the surgery. In contrast, the addition of diuretics increased creatinine clearance to 600–700 μl/min/100 g BW throughout the study. According to CKD Guidelines, stage 3 is defined as a 50–75% reduction in GFR. It could be implied from our extrapolations that subtotally nephrectomized animals had GFR reduced by 50% (approximately stage 3) and that the diuretics added to the combined RAS + ET_A_ blockade were able to increase it by 25%. In other words, the addition of a diuretic improved the stage 3 to stage 2 of experimental CKD.

Interestingly, the positive effects of a combination of diuretic chlorthalidone with ET_A_ receptor blockade on albuminuria were demonstrated in Dahl rats on a high-fat/high-salt diet, a model of metabolic syndrome, after 4 weeks of treatment (Jin et al., 2014). In the same experimental model, the combination of enalapril (angiotensin enzyme inhibitor) and atrasentan was used in animals kept on a high-salt diet for 12 weeks. While a combination of both drugs reduced cardiac hypertrophy, atrasentan (but not enalapril) attenuated proteinuria and serum creatinine level (Samad et al., 2015).

Although there was a tendency to lower BP in rats with additional diuretic treatment, the blood pressure and cardiac hypertrophy were similarly attenuated in all three groups of treated rats. These findings are not surprising as the maximal RAS blockade with losartan and trandolapril was employed in our study and hence the effective lowering of blood pressure to normotension was already achieved by RAS blockade alone. It seems that the beneficial renoprotective effects of diuretics on renal function (proteinuria and creatinine clearance) and renal damage (glomerular area and kidney weight together with tubulointerstitial changes) were achieved beyond those exerted by a normalization of blood pressure. Similar effects of different classes of RAS blockers (including direct renin inhibitor aliskiren) on BP normalization were also demonstrated in TGR rats without nephrectomy (Vaněčková et al., 2016).

Concerning the body weight, a diuretic added to the combined RAS + ET_A_ blockade significantly decreased body weight while no significant body weight gain was observed in rats with combined RAS + ET_A_ blockade. However, to determine whether edema is present, the volume and distribution of body fluids should be measured. Nevertheless, a significantly higher urine production in the diuretic-treated TGR with combined RAS + ET_A_ blockade was discerned at weeks 13, 40 and 50 (31 ± 2 vs. 24 ± 1 ml/24 h at week 13, 31 ± 2 vs. 25 ± 2 ml/24 h at week 40 and 27 ± 1 vs. 23 ± 1 ml/24 h at week 50) as compared with TGR treated only with RAS + ET_A_ blockade. Importantly, kidney weights (both absolute and relative) were greatly reduced in the rats treated with an additional diuretic. This finding together with a substantially smaller glomerular area in diuretic-treated rats is consistent with a glomerular hypertrophy concept (Brenner et al., 1996) suggesting that glomerular hyperfiltration and hypertension are major factors contributing to CKD development. Future studies are needed to elucidate whether podocytes are involved in this process (Kriz and Lemley, 2015).

It remains to be determined, whether the combination of other diuretics and RAS blockers would be similarly effective, since a combination of losartan with hydrochlorothiazide, but not with furosemide, was beneficial in rats with subtotal nephrectomy (Arias et al., 2013, 2016). Moreover, the benefits of hydrochlorothiazide against furosemide were demonstrated also in human studies (Fuwa et al., 2016). Although thiazides were previously not recommended in CKD patients, their use even in advanced stages of CKD is now being reconsidered (Sinha and Agarwal, 2015) because in combination with RAS blockade they were effective in reducing BP (Cirillo et al., 2014) and proteinuria (Morales et al., 2015). The superiority of losartan (ARB) has been documented not only in an earlier study in animals with subtotal nephrectomy (Fujihara et al., 2005) but also in a recent study (Maquigussa et al., 2018) showing the ability of losartan to restore anti-fibrotic molecules Klotho and PPAR gamma and thus to reduce tubulointerstitial fibrosis which is one of the key factors underlying the progression of CKD to ESRD (Zoja et al., 2006). In addition, newer thiazide-like diuretics (chlorthalidone and indapamide) might also be considered although their usage has not been supported by clinical data (Thomopoulos et al., 2015).

## Data Availability

The datasets generated for this study are available on request to the corresponding author.

## Ethics Statement

The animal study was reviewed and approved by Ethical Committee of the Institute of Physiology, Czech Academy of Sciences.

## Author Contributions

IV conceived and designed the project and wrote the manuscript. SH, HR, ZVa, and MK performed the experiments. ZVe examined the histology. IV and JZ analyzed the data and interpreted the results. JZ, LČ, and EK-J edited the manuscript. All authors read and approved the final version of the manuscript.

## Conflict of Interest Statement

The authors declare that the research was conducted in the absence of any commercial or financial relationships that could be construed as a potential conflict of interest.
